# Outcome of twin pregnancies conceived after assisted reproductive techniques

**DOI:** 10.4103/0974-1208.39593

**Published:** 2008

**Authors:** Baxi A, Kaushal M

**Affiliations:** Department of Obstetrics and Gynecology, Disha Fertility and Surgical Center, Indore, Madhya Pradesh, India

**Keywords:** Assisted reproductive technique, multiple pregnancies, spontaneous twin pregnancy

## Abstract

**CONTEXT:**

There is a continuous controversy regarding the obstetric perinatal outcome of twin pregnancies conceived after assisted reproductive techniques (ART). There is an ongoing discussion whether theses parameters may show poorer results as compared to spontaneous conception.

**AIMS:**

To evaluate the outcome of multifetal pregnancies and to compare maternal and neonatal complications between spontaneously conceived and assisted reproductive therapy.

**SETTINGS AND DESIGN:**

Prospective case-control study.

**MATERIALS AND METHODS:**

In this prospective case-control study of 2-year duration, obstetric and perinatal outcomes were compared in 36 ART twin pregnancies (Group A) with 138 twins who conceived naturally (Group B). The outcomes were analyzed and used for a comparison between spontaneous and assisted multifetal pregnancies.

**STATISTICAL ANALYSIS:**

The continuous variables were analyzed by Student's *t*-test and categorical variables were analyzed with Fisher's exact test.

**RESULTS:**

Pregnancy-related complications like pregnancy-induced hypertension, antepartum hemorrhage, were similar in both groups. Incidence of cesarean section, preterm delivery, and hospital stay was significantly more in Group A vs. Group B, *P* < 0.001. The newborns in the assisted group had more complications than the spontaneous group; most notable were respiratory distress syndrome, newborn intensive care admission, sepsis, and longer hospital stay (4.8 days vs. 1.6 days, *P* < 0.001).

**CONCLUSIONS:**

Increased rates of cesarean section and preterm delivery are the main reasons for increased obstetric risk in pregnancies conceived through ART. Preterm birth and neonatal prematurity-related complications were the main cause for longer stay in hospital in ART-conceived twins.

Assisted reproductive techniques (ART) and its variants have become routine infertility treatments in industrialized countries.[1] The Centers for Disease Control and Prevention (CDC) defines assisted reproductive technique (ART) more narrowly as any procedure that entails the handling of both eggs and sperm or of embryos for the purpose of establishing a pregnancy.[2] These procedures include *in vitro* fertilization (IVF) and intracytoplasmic sperm injection (ICSI). Approximately one million children worldwide have been born through ART. The incidence of pregnancies by ART (IVF and ICSI) is increasing over the years.[3,4] Incidence of birth following ART is also rising in India.

Studies have been done worldwide to analyze the outcome of multifetal pregnancy.[5,6] There is a lack of sufficient knowledge on outcome in IVF/ICSI pregnancy especially in India, so we went and analyzed our data.

Multiple gestation rates are high in assisted reproductive treatment cycles because of the perceived need to stimulate excess follicles and transfer excess embryos to achieve reasonable pregnancy rates. The course of pregnancies and the health of children born after assisted reproductive technologies are two of the most important outcome parameters of the quality of the techniques. Because the goal of infertility therapy is a healthy child and multiple gestations put that goal at risk. Therefore, perinatal outcome is measured by comparing ART twins with naturally conceived twins. The aim of this study was to determine the outcomes of multifetal pregnancies and to compare the outcomes between those that were spontaneously conceived and the ART multifetal pregnancies in a single institute of Indore (MP).

## SUBJECTS AND METHODS

Present study was done in a private infertility clinic and ARU of Indore (MP) for 2-year duration. Study period was from May 2004 to April 2006. Perinatal outcome was studied in 36 ART-conceived twins (IVF 19 and ICSI 17) and were compared with 138 pregnancy who conceived naturally in the study period. ART methods include IVF and ICSI. This ART center is attached to a obstetric unit in private hospital where deliveries were conducted. Controls were spontaneously conceived twin pregnancy who delivered at the same obstetric unit during the study period. Information about the obstetric and perinatal outcome of the control was obtained from hospital records and through the questionnaires sent to referring obstetrician. Twin pregnancies that initially conceived as triplets, quintuplets and where spontaneous fetal resorption or fetal reduction was done were excluded from the study. Also in the control group, those twin pregnancies where spontaneous fetal resorption occurred were excluded. As all ART pregnancies delivered at the same center, obstetric and neonatal follow-up was easier. Gestational age for ART-conceived pregnancy was calculated from the day of oocyte retrival and by the combination of last menstrual period and first dating ultrasound for the spontaneous pregnancy. Preterm labor was considered whenever labor occurred before 37 weeks of gestation.

Pregnancy-induced hypertension was defined as blood pressure >140/90 mm Hg noted on two or more occasions after 20 weeks of gestation in previously normotensive women with or without protienuria. Respiratory distress syndrome was defined as presence of characteristic radiographic finding and requirement of oxygen at 24 h. Sepsis was diagnosed based on clinical criteria and laboratory tests. Birth weight discordance was defined as weight difference of more than 20%. Detailed analysis of pregnancy complications, birth events, maturity, and birth weights were taken into account. All neonates were evaluated and care given by a expert neonatologist. Neonates followed up till 4 weeks following date of birth.

We compared the following variables between the two groups: maternal age, gravidity, parity, pregnancy complication, gestational age at delivery, mode of delivery, birth weight, congenital and chromosomal abnormalities, sepsis, respiratory distress syndrome, and neonatal intensive care unit (NICU) stay. The institutional ethics committee approves the study.

The final data were double entered using EpiInfo version 6. Analysis was performed by using STATA version 7. The maternal and fetal outcomes were compared between the spontaneous and ART groups. The continuous variables were analyzed by Student's *t*-test and categorical variables were analyzed with Fisher's exact test. Statistical significance was defined as a probability value of *P* < 0.05.

## RESULTS

During the study period of 2 years, of the 196 twin pregnancies recorded in the hospital, 174 twin pregnancies were taken into account after exclusion criteria. Of the 174 twin pregnancies, 36 women conceived by ART (including IVF and ICSI) and 138 women had spontaneously conceived twin pregnancies. Table 1 lists characteristics of pregnant women according to the method of conception. The women conceived by ART were older than those who conceived spontaneously (28.8 ± 4.21 vs. 27.2 ± 3.84), but the difference was not statistically significant. There was a significant difference in gravida and parity of women in ART group and women with spontaneous pregnancies (1.21 ± 0.52 vs. 2.12 ± 1.33 and 0.21 ± 0.3 vs. 0.72 ± 0.9, *P* < 0.001).

**Table 1:** Maternal characteristics in assisted reproductive technique and spontaneous twin conception

	ART, *n* = 36	Spontaneous, *n* = 18	*P* value mean
Age ± SD (years)	28.8 ± 4.21	27.2 ± 3.84	<0.05
Gravidity	1.21 ± 0.52	2.12 ± 1.33	0.001
Parity	0.21 ± 0.3	0.72 ± 0.9	<0.001

ART = Assisted reproductive techniques, SD = standard deviation

Table 2 shows obstetric and neonatal outcome among twins. Pregnancy-related complications like antepartum hemorraghe, pregnancy-induced hypertension, gestational diabetes, and postpartum hemorrhage were similar in both the groups and were not statistically significant. Preterm labor (88.9% vs. 57.9%) was more common in study group (*P* < 0.05). Mean gestational age at the time of delivery was less in ART twin pregnancy than spontaneous pregnancies (34.51 ± 3.1 vs. 36.81 ± 2.5). A significant difference was seen between both groups with respect to the mode of delivery [Table 3]. The cesarean birthrate in ART group was significantly higher than that of spontaneous group (88.4% vs. 94.4%, *P* < 0.001). The mean birth weight in ART twin pregnancy was significantly lower than spontaneous conception (1627 g + 151 vs. 2421 g + 681). Although birth weight at particular gestational age was corresponding [Table 4], significant difference in birth weight could be attributed to lower gestation age at the time of birth.

**Table 2:** Pregnancy complication in ART and spontaneously conceived twin pregnancy

	ART, *n* = 36	Spontaneous, *n* = 138	*P* value
Antepartum hemorrhage	4 (11.1)	17 (12.3)	ns
Pregnancy-induced	8 (22.2)	28 (20.9)	ns
hypertension			
Gestational diabetes mellitus	2 (5.6)	5 (3.6)	ns
Premature rupture of	4 (11.1)	14 (10.1)	ns
membranes			
Preterm labor	32 (88.9)	80 (57.9)	<0.001
Intrauterine growth	17 (51.5)	45 (32.6)	<0.05
restriction			

Figures in parentheses are in percentage

**Table 3:** Neonatal characteristics of assisted reproductive technique and spontaneous twin delivery

	ART, *n* = 36 (%)	Spontaneous, *n* = 138 (%)	*P* value
Mode of delivery			
Vagina	l2 (3.6)	16 (11.6)	
Abdominal	34 (94.4)	122 (88.4)	<0.001
Mean (+SD) gestational			
Age at delivery	34.51 ± 3.1	36.81 ± 2.5	<0.05
Range (weeks)	26-38	27-39	
Mean birth weight			
At birth (g)	1627 ± 151	2421 ± 681.5	<0.001
Range (g)	920-2860	1080-2970	
Mean NICU Stay (days)	4.8	1.6	<0.001
Sepsis	2 (5.6)	10 (7.0)	ns
Congenital malformation	1 (2.8)	3 (2.1)	ns
Respiratory distress	1 (2.8)	5 (3.6)	ns
syndrome			

SD= Standard deviation

**Table 4:** Birth weight at gestational age

Gestational age (weeks)	ART twin (g)	Spontaneous twin (g)
<28	905	987
28-32	1376	1479
32-36	1904	2204
>36	2306	2445

The newborns in the assisted group had more complications than the spontaneous group; most notable were respiratory distress syndrome, sepsis, and longer newborn intensive care admission. However no statistical significance was found between the two groups except for longer hospital stay (6 days vs. 15 days, *P* < 0.001. Respiratory distress syndrome and sepsis in both the groups were more common in twins who delivered before 34 weeks of gestation. Average gestational age for RDS and sepsis in ART group is 29.8, 28.4 weeks and in spontaneously conceived twin 30.0 and 29.3 weeks. The figure for congenital malformation was comparable between the two groups. Neonatal follow-up after 4 weeks of delivery was comparable in terms of growth chart between the two groups.

## DISCUSSION

There are continuous controversies regarding the perinatal outcome of twin pregnancy conceived after ART. One study showed that ART-associated twins have a lower perinatal mortality than spontaneously conceived twins.[7] However, another study showed that ART twins are more likely to result in discordant and low birth weight.[8] In the present study, we found that there is increased incidence of preterm delivery, cesarean delivery, and more NICU stay. These results were in accordance with outcome of some previous studies.[5,6,9–10] Moise *et al*. found that twins conceived by IVF are at significantly higher risk for prematurity and associated neonatal morbidity and mortality than spontaneously conceived twins.[5] Similarly Daniel *et al*, found that ART-conceived twin pregnancy is at greater risk than non-ART conceived one for pregnancy complications and adverse perinatal outcome.[6]

Several factors may attribute to this adverse perinatal outcome in ART pregnancy. These are hyper stimulation of endometrium, diseased tubes, advancing age, etc. In our study, there was no significant difference in the mean age of the two groups, but the proportion of primiparous women was significantly higher in ART group. Several case control studies observed adverse outcome for ART-conceived pregnancy, where age and parity was matched, which suggest that infertility status and ART procedures may have an adverse outcome in these women.[11]

Brian *et al*. observed that ART-associated twins have lower perinatal mortality than spontaneously conceived twins.[7]

Some of the previous studies reported that ART-conceived twins has comparable perinatal outcome as with naturally conceived twins.[11,12] Koudstaal *et al*. observed similar perinatal outcome of ART twin pregnancy and those who conceived naturally or after ovulation induction. But in this study deliveries prior to 28 weeks were excluded.[12]

In our study although the rate of preterm birth and stay in intensive neonatal care unit was higher than the control group, but eventually the perinatal outcome was comparable to that of spontaneously conceived twin pregnancies. These findings are in agreement with some previous studies.[5,6]

Preterm birth is a frequent problem in women who undergo treatment for infertility. Infertile women seem to have predisposition to giving preterm birth and low birth weight babies.[13,14] Even singleton births resulting from ART are associated with an increased risk of low birth weight.[15]

Increased hospital stay of the mother and baby as noticed in our study could be explained on ground of increased preterm birth and neonatal prematurity-related complications.

The overall cesarean delivery rate in the present study was high with the ART group having a higher rate than spontaneous group. Increased operative delivery in twin pregnancy has been defined in many studies.[15,16] As there is more malpresentations in the second twin and there is also evidence that the risk of neonatal morbidity and mortality in the second twin is higher if delivered vaginally. Obstetricians lean toward cesarean delivery in twin pregnancy.[17] Obstetricians anxiety and their concern while managing pregnancies in an infertile women may increase the rate of cesarean section in this group of patients. Although studies have suggested that the outcomes were not improved by cesarean section.

Obstetric complications like pregnancy-induced hypertension, preterm rupture of membranes, antepartum hemorrhage, gestational diabetes, and postpartum hemorrhage were not different between the two groups. Luke *et al*. reported that assisted conception was not normally by itself a risk factor adverse outcome.[18] Although in our study pregnancy complications in ART pregnancies are comparable with non-ART twin pregnancies, the ART twin mothers were more likely to be on sick leave or hospitalized during pregnancy. This could be because of increased anxiety and concern for the newborn.

Studies are done to evaluate the safety of ART procedures by collecting data on neonatal outcome and congenital malformations during pregnancy and birth. Anja *et al*. reported similar risks of neurological squeal twins from assisted conception as their naturally conceived peers. Also physical health and growth of IVF/ICSI twins were comparable with that of non-IVF/ICSI twin.[19]

Limitations of this study were sample size, matching criteria, and choice of control group. Also many of the spontaneously conceived twin pregnancy had received antenatal care by different obstetricians; therefore, there was some difference in obstetric policy that is the major methodical problems in similar studies.

In conclusion our findings showed that increased obstetric risk in pregnancies conceived through ART is due to increased rates of preterm delivery and cesarean sections and longer hospital stay. In the present study, we were not able to analyze the outcome in term of chorionicity as it could not be determined in control group. Also the long-term neurological development of twins could not be done because of lack of compliance. Although findings of the study will help obstetricians while counselling patients seeking ART with respect to the anticipated outcome of twin pregnancy.

To make ART the standard of care in the treatment of infertility, there is a need for more clinical studies, intensive counselling of patients, and an increased sense of responsibility in the health care providers.
